# Effect of daily versus weekly home fortification with multiple micronutrient powder on haemoglobin concentration of young children in a rural area, Lao People's Democratic Republic: a randomised trial

**DOI:** 10.1186/1475-2891-10-129

**Published:** 2011-11-24

**Authors:** Sengchanh Kounnavong, Toshihiko Sunahara, C G Nicholas Mascie-Taylor, Masahiro Hashizume, Junko Okumura, Kazuhiko Moji, Boungnong Boupha, Taro Yamamoto

**Affiliations:** 1 National Institute of Public Health, Ministry of Health, Vientiane, Lao People's Democratic Republic; 2 Department of International Health, Institute of Tropical Medicine (NEKKEN) and the Global Centre of Excellence Program (GCOE), Nagasaki University, Japan; 3 Division of Biological Anthropology, Department of Archaeology and Anthropology, University of Cambridge, United Kingdom; 4 Research Institute for Humanity and Nature, Kyoto, Japan

**Keywords:** Anaemia, multiple micronutrient powder, supplementation, home fortified food, Lao PDR

## Abstract

**Background:**

Multiple micronutrient deficiencies, in particular iron deficiency anaemia (IDA) is a severe public health problem in Lao People's Democratic Republic (Lao PDR). Because of the practical difficulties encountered in improving the nutritional adequacy of traditional complementary foods and the limitations associated with the use of liquid iron supplementation for the treatment and prevention of IDA in infants and young children, recently, home-fortification with multivitamins and minerals sprinkles was recommended. This study aims to compare the effect of twice weekly versus daily supplementation with multivitamins and minerals powder (MMP) on anaemia prevalence, haemoglobin concentration, and growth in infants and young children in a rural community in Lao PDR.

**Methods:**

A randomized trial was conducted in six rural communities. Children aged 6 to 52 months (n = 336) were randomly assigned to a control group (n = 110) or to one of two intervention groups receiving either two sachets per week (n = 115) or a daily sachet (n = 111) of MMP for 24 weeks; 331 children completed the study. A finger prick of blood was taken at baseline, at week 12, and again at week 24 to determine haemoglobin concentration. Anthropometric measurements were taken every 4 weeks. The McNemar test was used to assess within group differences at three time points in the study subjects with anaemia and one-way ANOVA was used to assess changes in mean haemoglobin concentration in the treatment groups.

**Results:**

MMP supplementation resulted in significant improvements in haemoglobin concentration and in the reduction of anaemia prevalence in the two treatment groups compared with the control group (*p *<0.001). The severely to moderately anaemic children (Hb <100 g/L) on daily supplementation recovered faster than those on twice weekly supplementation. MMP was well accepted and compliance was high in both treatment groups. Overall, the improvement in the weight for age Z-score was very small and not statistically significant across the three study groups.

**Conclusions:**

MMP supplementation had positive effects in reduction of anaemia prevalence and in improving haemoglobin concentration. For severely to moderately anaemic children, daily MMP supplementation was more effective in improving haemoglobin concentration and reducing anaemia prevalence. A longer intervention period is probably needed to have a positive effect on growth.

## Background

According to UNICEF/WHO/WFP, micronutrient or vitamin and mineral deficiencies affect approximately 2 billion people worldwide [1]. The adverse effects of micronutrient deficiencies during childhood are substantial. Micronutrient deficiencies have negative effects on growth and development, and cause reduced psychomotor performance, and increased morbidity and mortality [2-8].

The main cause of the multiple deficiencies is a poor quality diet, often as the result of an inadequate intake of animal sources of foods. Additionally, non-nutritional factors such as parasitic infections, genetic haemoglobinopathies, malaria, and infectious diseases impair nutritional status and health and alter the metabolism of multiple micronutrients [9]. Infants and young children are particularly at risk of micronutrient deficiencies. From the age of 6 months, the nutrient requirements of infants need to be met by breast milk and complementary foods. Although possible modifications to the traditional recipes, such as adding extra animal source foods and reducing absorption-inhibiting components such as phytate, were considered, the diet would still be unlikely to meet the requirements for micronutrients such as iron, calcium, and zinc. Additionally animal source foods alone may not provide enough vitamins and minerals for young children [10,11]. Therefore, in developing countries, micronutrients are very likely to continue to be limited in the diets of young children between the ages of 6 and 23 months. This problem also applies in Lao People's Democratic Republic (Lao PDR) where the quality of complementary food, that is predominantly cereal-based, is very poor[12]. Thus, for children aged 6-23 months, vitamins and minerals need to be added to their diets to improve development and growth and reduce morbidity and mortality[9].

Anaemia, which is mainly due to iron deficiency, is one of the major micronutrient deficiencies in developing countries. In the Lao PDR Multiple Indicator Cluster Survey 2006 and the Lao PDR National Nutrition Survey 2006, the prevalence of anaemia in children 6-59 months of age was reported to be 41%. The prevalence was much higher (63.5%) in children aged 6-23 months [13]. According to the World Health Organization (WHO), over 40% prevalence of anaemia indicates a very severe public health problem [14].

Because of the practical difficulties encountered in attempts to improve the nutritional adequacy of traditional complementary foods [15] and the limitations associated with the use of liquid iron supplementations for the treatment and prevention of iron deficiency anaemia (IDA) in infants and young children[16] and because multiple deficiencies coexist with IDA, home fortification with multivitamins and minerals in a powdered form, known as sprinkles, has been widely promoted as a way to address IDA and micronutrient deficiencies [17-25]. One of the main benefits of sprinkles is that they can be easily incorporated into the currently recommended complementary feeding practices for infants after 6 months of age and can therefore contribute to healthy infant weaning practices [26].

However, the effect of adding sprinkles to home-prepared foods may depend on the local food culture and acceptance by local people. Therefore, the multivitamins and minerals powder needs to be tested locally to clarify the optimal starting point, the duration of use, and its acceptability [27]. A previous study in young Vietnamese children found that weekly supplementation with multiple micronutrients was as effective as daily supplementation in improving haemoglobin concentration and proposed that weekly instead of daily supplementation was cheaper and program compliance may be better [28].

The primary aim of the present study was to compare the effects of multivitamins and minerals powder (MMP) given as two sachets on 2 separate days during the week with one sachet given daily on haemoglobin concentration, the prevalence of anaemia, and growth in infants and young children. We also aimed to assess MMP compliance for the two regimens and the acceptability of the product among the mothers or caretakers of the study subjects.

## Methods

### Study area

This study was conducted in six communities in the Lahanam zone, Songkhone District, Savannakheth Province, 600 km south of the capital city, Vientiane, Lao PDR. The Health and Demographic Surveillance System (HDSS) was established in this area in 2004 and all the population is registered.

The Lahanam area has a relatively high production of rice, cotton and watermelon, and most villagers have access to adequate amounts of rice. Other foodstuffs are seasonally available from nearby small patchy forests and from the market town (within 10 km of the Lahanam area). Intake levels of fat, calcium, iron, and retinal in the area were found to be very low compared with the recommended dietary allowance of Thailand and the WHO/FAO [29].

The first complementary food that is used in the area is principally glutinous rice, which is given pre-chewed by mothers to nearly all infants in the first week of their life. This traditional early use of complementary food may decrease or stop the infant's intake of breast milk [30].

There is only one health centre in the area providing primary health care. This centre serves a population of 7,000 through six auxiliary nurses and an average of two village health volunteers (VHVs) in each village. A malaria control program was successfully executed in all the villages 10 years ago. Each household was categorized into one of two socioeconomic status groups: high (with electricity, improved water source and latrine) and low (lacking one or all of these).

### Study design and randomization

The study was a randomized trial. A statistical power analysis (80% power and significance of *p *= 0.05) showed that to reduce anaemia by 20% (from 41% to 21%) and to improve haemoglobin concentration by 0.56 g/L, and to compensate for 10% loss to follow-up, a sample size of 110 children would be needed in each group.

In an effort to promote community participation, a series of meetings with local authorities (heads of the villages), parents, VHVs, and health centre staff were held before commencement of the study to explain the study objectives. Individual written informed consent was obtained at the time of enrolment from the mothers or legal guardians of all the children involved in the study. The study was approved by the Nagasaki University Ethical Review Board (Japan) and the National Ethic Committee for Health Research of the Ministry of Health in Lao PDR.

From the HDSS database of the National Institute of Public Health (NIOPH), 367 eligible pre-school age children were identified. Inclusion criteria were: (i) age 6 to 53 months at the time of recruitment; (ii) willingness to participate; (iii) receiving complementary food in addition to breast milk; and (iv) apparently healthy. Exclusion criteria were: (i) having fever or any illnesses on the day of enrolment; (ii) baseline level of haemoglobin less than 70 g/L; and (iii) currently receiving iron supplementation. Of the original 367 children who met the criteria, 17 were absent at the time of enrolment, and 14 were excluded because they had infections with fever on the day of enrolment. Therefore, a total of 336 children were enrolled in the study.

Before recruitment began, we used a simple computer program (a random number generator) for the randomization process, which was done by household. We then enrolled the 336 eligible children and randomly allocated them to three groups: a control group (n = 111), a group given twice weekly multiple micronutrients supplementation (n = 115), and a group given daily multiple micronutrients supplementation (n = 111). Children in families with two or more children who fulfilled the requirements to participate in the study were all included and treated as separate cases; however, all the children in one family were allocated to the same group. There were 215 one-child families, 49 families with two children, and three families with four children in the study.

As part of the routine government health services, all children below the age of five years received a single high dose of vitamin A every 6 months, and those aged 24 months or older received a single dose of mebendazole for deworming in the 2 months prior the study. Children who had not received mebendazole were given it during the baseline survey.

Over 24 weeks, the daily group was given MMP supplementation from Monday to Sunday (7 d/wk), and the twice weekly group was given MMP supplementation on Monday and again on Friday. Because of technical and financial constraints, placebo could not be produced locally, so following the normal standard of care in Lao PDR at the time of the study; the control group received the 6-monthly high-dose vitamin A supplementation instead of a placebo. After completing this study, all subjects in the control group received 60 sachets of MMP.

### Micronutrient supplements

The MMP supplement used in this study was MixMe™ manufactured by DSM Nutritional Products Europe, Ltd., CH 4002 Basel. The nutrients and amounts used in the multi-micronutrient formula are based on the recommendations by UNICEF/WHO/WFP for one recommended dietary allowance of 15 vitamins and minerals. The nutrient content of 1 g of MMP was vitamin A (RE 400 μg), vitamin D3 (5 μg), vitamin E (TE 5 mg), vitamin B1, B2, B6 each (0.5 mg), folic acid (150 μg), niacin (6 mg), vitamin B12 (0.9 μg), vitamin C (30 mg), iron (10 mg), zinc (4.1 mg), selenium (17 μg), copper (0.56 mg), and iodine (90 μg). The MMP was supplied in a single-dose sachet (1 dose = 1 sachet) and one pack contained 30 × 1 g sachets.

The doses of micronutrients used in this study were calculated based on the WHO recommendation on the dosage schedules for iron supplementation to prevent IDA [14]. Indications for supplementation are when the diet does not include foods fortified with iron or when anaemia prevalence is above 40%. It is recommended that children from 6 to 23 months of age should receive 2 mg of iron per kg body weight per day and children from 24 to 59 months of age should receive the same dose, up to a maximum of 30 mg per day, for 3 months.

The dose of iron in the MMP used in this study was 10 mg, which is within the range of daily recommended dosage of iron for young children according to the American Food and Drug Administration. A total of 168 sachets were provided to the daily supplementation group and 48 sachets were given to the twice weekly supplementation group.

### Data collection

The study commenced in February 2009 with focus group discussions and in-depth interviews with mothers and field promoters (VHVs and members of the Lao Women's Union) to gather information on current child feeding practices and on how to introduce and promote MMP supplementation for children. This phase was completed in March 2009 with a baseline survey in all six communities. Mothers or legal guardians were invited to a central setting such as a health centre or temple in each of the villages, and interviews were conducted by the medical staff from the NIOPH. Information was gathered on; (i) family characteristics including mother's education level, working status (working outside or at home), ownership of latrine, and access to improved water sources; (ii) feeding practices, noting the age of starting complementary foods, the type of complementary foods, and number of meals consumed per day; (iii) knowledge of anaemia; and (iv) medical history (occurrence of diarrhoea or cough in the previous 2 weeks and receipt of routine deworming treatment). After this, the MMP supplementation intervention began, lasted for 24 weeks, and ended in October 2009.

### Assessment of haemoglobin concentration

At weeks 0, 12 and 24 of the study, capillary blood samples were obtained from a finger prick using aseptic technique; haemoglobin concentrations were measured immediately with a portable battery-operator Hemocue B-Haemoglobin photometer (Hemocue Inc., Angelholm, Sweden) by trained technicians [31], who were unaware of the allocation of micronutrient supplement. Anaemia was defined as a haemoglobin concentration below 110 g/L.

### Anthropometric measurement

The height and weight of the infants and children were measured every 4 weeks during the 24-week intervention. A calibrated SECA scale with intervals of 0.1 kg was used to measure weight. An infant length board with a sliding foot board was used to measure the recumbent length to the nearest 0.1 cm of children less than 24 months, and a wooden scale with a sliding head piece was used to measure the standing height of children 24 months and older. Two field workers, who were unaware of which group a child belonged to, completed the measurements in duplicate using standardized WHO procedures[32]. Height for age, weight for age, and weight for height Z-scores were calculated using the WHO Child Growth Standard (WHO Anthro software, version 3.01) [33].

### Training of village health volunteers

A total of 16 VHVs were recruited and given a 1-day training session before the initiation of the study. The benefits, adverse side effects, usage of the MMP supplement (food demonstration), MMP supplement nutrition education and instructions on follow-up using the monitoring form were all explained. The VHVs delivered MMP supplements to the intervention groups on a weekly basis and instructed the mothers on how to administer the dose in a single meal, twice weekly for one group (the TWS group) and daily for the other (the DS group). To ensure that children consumed the entire dose, mothers were asked to mix the MMP supplement with a small amount of the child's food just before consumption.

### Monitoring forms (compliance and acceptability)

The VHVs used monitoring forms to record the number of MMP sachets consumed by the children in the two intervention groups, any side effects, and any illnesses that occurred during the study period. For the TWS group, a VHV visited the house every Monday providing one sachet and every Friday giving the other sachet. For the DS group, VHVs visited the house every Monday and provided all seven sachets. The sachets left unconsumed from the previous week were counted on the next Monday's visit. The three health centre nurses collected the monitoring forms weekly from the 16 VHVs. All monitoring forms were checked by the NIOPH supervisor and reviewed by the main researcher on a monthly basis. The total number of empty sachets was used to measure compliance. For the TWS group, good compliance was defined as when children consumed more than 70% of all the MMP sachets provided, and for the DS group when at least five sachets of the MMP supplements provided per week were consumed during the 24 weeks of the study.

### Statistical analysis

Data were analysed using the PASW statistical package, version 18.0 (SPSS Inc., Chicago, IL, USA). Socio-demographic, health, and nutrition characteristics of the study subjects were summarised as mean and standard deviation (SD) for continuous variables and as frequency for categorical variables. Differences in prevalence were tested with the Pearson chi-squared test. Differences in mean haemoglobin concentration and Z-scores between the groups at the beginning and at the end of the intervention were examined using Repeated Measures Analysis of Variance. The McNemar test was used to assess within group differences at three time points in the study subjects with anaemia and one-way ANOVA was used to assess changes in mean haemoglobin concentration in the treatment groups. All the analyses were carried out for all subjects and separately for the children who were anaemic at baseline. Values of *p *<0.05 were considered to be significant for all tests.

## Results

**Figure 1:**
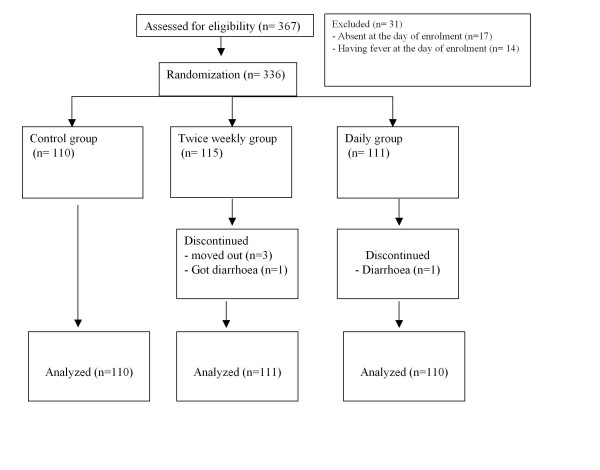
**Enrolment procedure**.

Of the 336 children recruited, five (1.5%) were lost to follow-up before the 12-week assessment (three moved out of the study area, and two developed diarrhoea and their mothers refused to continue the study). Thus 331 study subjects completed all 24 weeks of the trial (Figure 1).

### Baseline characteristics

Baseline information (Table 1) revealed some typical characteristics of population in the rural communities of Lao PDR. The majority of study subjects had mothers with relatively low levels of education and were from households with low access to basic facilities such as improved water sources and latrines. The nutritional status of the study subjects was characterized by a relatively high prevalence of both stunting and underweight. There were no significant differences between the groups in terms of sex distribution, mean age, weight, and height.

**Table 1:** Baseline characteristics of the control, daily supplementation, and twice weekly supplementation groups

	Control	Twice weekly	Daily	P^1^
	(n = 110)	(n = 111)	(n = 110)	
Age, months	31.1 ± 14.6ª	32.4 ± 14.6	32.0 ± 13.3	0.793
Sex, male [% ]	34.5	46.8	42.7	0.168
Maternal age, year	28.4 ± 6.8	29.5 ± 8.4	29.0 ± 7.1	0.583
Maternal education, year	6.74 ± 3.1	6.38 ± 3.2	6.35 ± 3.6	0.637
Mothers working outside [% ]	93.6	84.7	90.9	0.080
High SES^2^[%)]	31.8	30.6	31.8	0.976
Household having > 6 members [% ]	49.1	49.5	58.2	0.314
Breastfeed infants[%]	33.6	23.4	20.9	0.074
Commercial baby formula [%]	19.1	13.5	19.1	0.473
Complementary food: Rice [%]	60.9	59.5	58.2	0.473
Meat [%(]	77.3	64.9	61.8	0.034
Egg [%(]	50.0	57.7	53.6	0.521
Haemoglobin concentration, g/L	114.3 ± 15.2	105.1 ± 13.3	107.1 ± 13.0	< 0.001
Anaemia, [% ]	34.5	58.6	53.6	< 0.001
Weight, kg	11.0 ± 2.5	11.3 ± 2.5	11.3 ± 2.5	0.637
Height, cm	84.2 ± 10.6	85.9 ± 10.8	85.6 ± 10.4	0.441
Weight-for-height Z-score	-0.48 ± 1.01	-0.63 ± 1.12	-0.53 ± 0.98	0.544
Height-for-age Z-score	-1.84 ± 1.62	-1.65 ± 1.70	-1.73 ± 1.53	0.677
Weight-for-age Z-score	-1.38 ± 1.13	-1.38 ± 1.16	-1.36 ± 1.10	0.990
Wasting^3 ^[%	4.5	9.9	7.3	0.307
Stunting^4 ^[%]	44.5	42.3	40.4	0.792
Underweight^5 ^[% ]	27.3	30.6	27.3	0.815

^a^Mean ± standard deviations (SD) (all such value, except where indicated otherwise)

^1^Based on ANOVA (continuous variables) or Chi-square tests (categorical variables)

^2 ^Socio-economic status "high" is defined as household with electricity, improved water source and latrine and "low" is lacking one or all of these.

^3^Wasting: Percentage below -2 standard deviations (SD) for weight-for-length/height Z-scores based on WHO child growth standard

^4^Stunting: Percentage below -2 standard deviations (SD) for length/height-for-age Z-scores based on WHO child growth standard

^5^Underweight: Percentage below -2 standard deviations (SD) for weight-for-age Z-scores based on WHO child growth standard

Although it was unintended, the haemoglobin concentration was significantly different at baseline in the control (n = 110) compared with in the two supplementation groups. Children in the control group had, on average, a higher mean haemoglobin concentration and thus a lower incidence of anaemia compared with the children in the two supplementation groups.

### Effects of the intervention

Overall, the prevalence of anaemia decreased from 58.6% to 26.1% in the TWS group and from 53.6% to 18.2% in DS group over the 24-week study period. Decrease in prevalence was also observed in the control group, from 34.5% to 23.6%; however, the change was significant only between weeks 12 and 24, whereas in the TWS and DS groups the changes were significant between the baseline and week 12 and between weeks 12 and 24. Thus, anaemia prevalence in the DS group was reduced by 35.4% in the 6 months compared with 32.5% in the TWS group (*p *= 0.043) and 10.9% (*p *<0.001) in the control group. To eliminate the effect of the initial difference in anaemia prevalence, we grouped the subjects who were anaemic separately from those who were not anaemic at baseline and compared the change in prevalence (Table 2). Of the children who were anaemic at baseline, the proportion that remained anaemic decreased most rapidly in the DS group: to 59.3% by week 12 and to 32.2% by week 24. The decrease in anaemia prevalence was lower in the TWS group (44.6% by week 24) and lowest in the control group (65.8% at week 24). By week 12, the proportion of anaemic children was significantly different between the control and DS groups and, by week 24, the proportion in the two intervention groups was significantly lower than in the control group. Although some children in the control and DS groups who were non-anaemic at baseline became anaemic in the early stages of the study, all but two of them were non-anaemic by week 24.

**Table 2:** Number (percentage) of children with anaemia at Week 12 and Week 24 of supplementation by treatment groups

	Non-anaemic at baseline			Anaemic at baseline		
	
	Control	Twice weekly	Daily	Control	Twice weekly	Daily
	(N = 72)	(N = 46)	(N = 51)	(N = 38)	(N = 65)	(N = 59)
Week 12	3 (4.2%)^A^	0 (0.0%)^A^	6 (11.8%)^B^	32 (84.2%)^A^	48 (73.8%)^A^	35 (59.3%)^B^
						
Week 24	1 (1.4%)	0 (0.0%)	1 (2.0%)	25 (65.8%)^A^	29 (44.6%)^B^	19 (32.2%)^B^
						

^A, B ^Within each class of anaemic status at baseline, groups with different letters have significant differences (McNemar Test, P < 0.05).

### Haemoglobin concentration

During the whole study period, the mean haemoglobin concentration increased from 107.1 g/L ± 13.0 g/L (SD) to 120.0 g/L ± 11.6 (SD) g/L in the DS group, and from 105.1 g/L ± 13.3 g/L (SD) to 118.0 ± 13.9 g/L (SD) in the TWS group. In the control group, the change in haemoglobin concentration was smaller, from 114.3 g/L ± 13.0 g/L (SD) to 117.4 ± 12.8 g/L (SD). Again, to eliminate the effects of the initial differences among groups, we separated the children by the haemoglobin concentration at baseline into non-anaemic (Hb >110.0 g/L), mildly anaemic (Hb 100.0-109.0 g/L), and severely to moderately anaemic (Hb <100.0 g/L).

**Figure 2:**
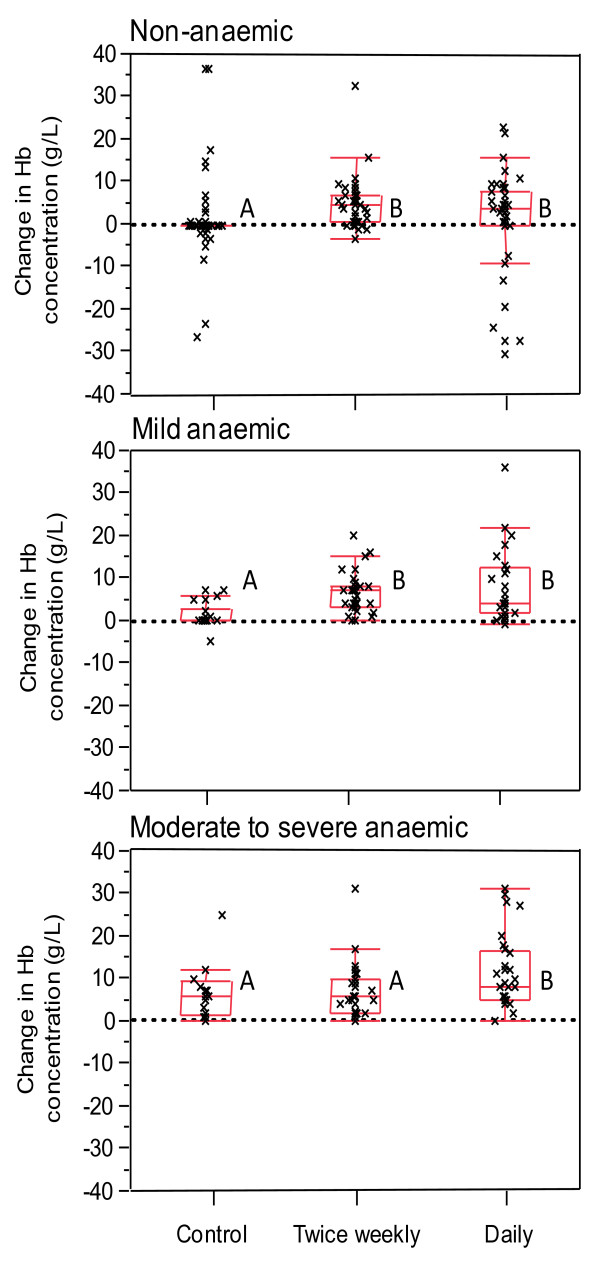
**Change in haemoglobin concentration from Week 0 to Week 12 in children who were non-anaemic (haemoglobin concentration ≥ 110 g/L), mild anaemic (100-109 g/L), and moderate to severe anaemic (< 100 g/L) at Week 0**. The box indicates the range of the 1st to 3rd quartile with central line at median; Vertical bars indicate the 1.5 times inter-quartile range outside 1st and 3rd quartiles. Within each panel, groups with different letters have significant difference (Mann Whitney U test, P < 0.05).

**Figure 3:**
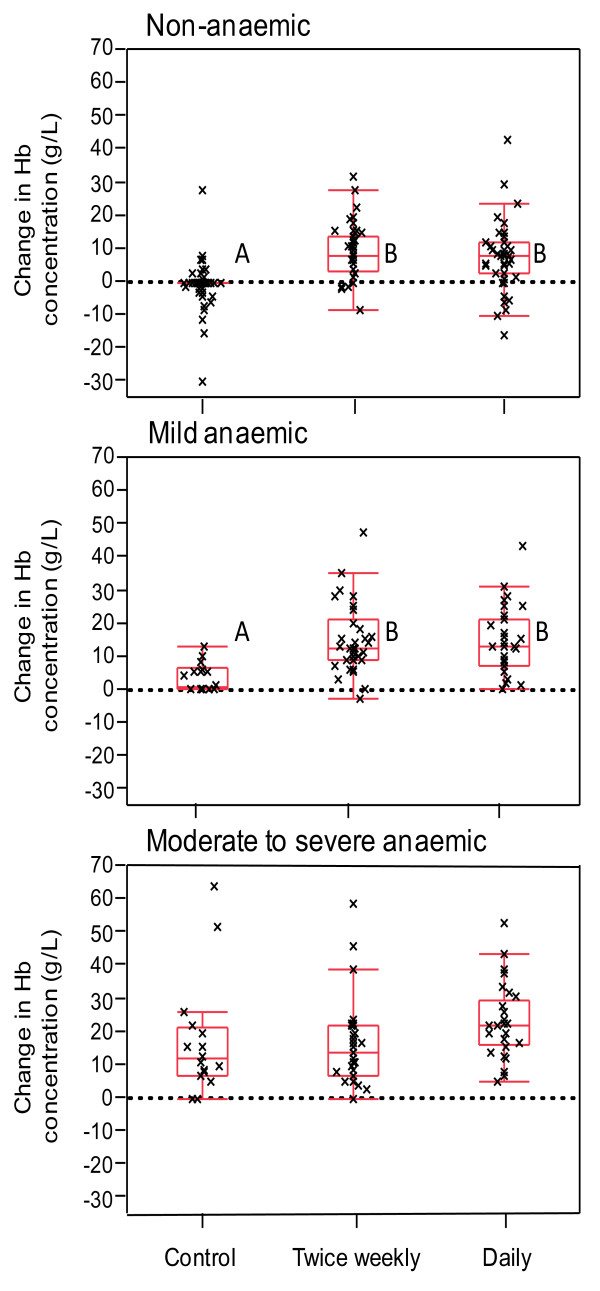
**Change in haemoglobin concentration from Week 0 to Week 24 in children who were non-anaemic (haemoglobin concentration ≥ 110 g/L), mild anaemic (100-109 g/L), and moderate to severe anaemic (< 100 g/L) at Week 0**. The box indicates the range of the 1st to 3rd quartile with central line at median; Vertical bars indicate the 1.5 times inter-quartile range outside 1st and 3rd quartiles. Within each panel, groups with different letters have significant difference (Mann Whitney U test, P < 0.05).

By week 12, the children in both the TWS and DS groups who were non-anaemic or mildly anaemic at baseline showed similar increases in haemoglobin concentration and this was significantly greater than for the non-anaemic or mildly anaemic children in the control group (Figure 2). However, for the severely to moderately anaemic children at baseline, the increase in haemoglobin concentration was greater in the DS group compared with in the TWS or control groups (Figure 2). The change in haemoglobin concentration by week 24 showed a similar trend; however, the differences between the groups were not significant because of the large variance in the control and TWS groups (Figure 3).

### Anthropometric measures

The anthropometric Z-scores for the three groups are shown in Table 3. Overall, the Z-scores of height for age increased, the Z-scores of weight for age showed no major improvements, and the weight for height Z-scores decreased in all groups. The increase in height for age Z-scores and the decrease in weight for height Z-scores were significantly greater in the control and DS groups than in the TWS groups.

**Table 3:** Change in anthropometric measurements in the three groups during the study period.

		Control	Twice-weekly	Daily	P^b^
		(n = 110)	(n = 111)	(n = 110)	
Weight for height Z score	Baseline	-0.48 ± 1.01^a^	-0.63 ± 1.12	-0.53 ± 0.98	0.544
	Week 24	-1.09 ± 1.08	-0.97 ± 1.06	-1.15 ± 1.04	0.414
	Change	-0.61 ± 0.96 ^A^	-0.33 ± 0.97 ^B^	-0.62 ± 0.84 ^A^	0.036
					
Height for age Z score	Baseline	-1.84 ± 1.62	-1.65 ± 1.70	-1.73 ± 1.53	0.677
	Week 24	-0.89 ± 1.56	-1.00 ± 1.49	-0.77 ± 1.41	0.532
	Change	0.95 ± 0.97 ^A^	0.65 ± 0.93 ^B^	0.95 ± 0.99 ^A^	0.029
					
Weight for age Z score	Baseline	-1.38 ± 1.13	-1.38 ± 1.16	-1.36 ± 1.10	0.990
	Week 24	-1.27 ± 1.08	-1.24 ± 1.10	-1.23 ± 0.93	0.969
	Change	0.11 ± 0.50	0.14 ± 0.45	0.12 ± 0.48	0.901

ª Mean ± SD (all such value, except where indicated otherwise)

^b ^Analysis of variance;

^A, B ^Differences between groups are significant when letters are different (t test, P < 0.05).

### Compliance and acceptability

All children in TWS group consumed two sachets of sprinkles per week, giving 100.0% compliance for this group. In the DS group, 72.7% of children consumed five or more sachets of MMP per week and 43.6% of the children consumed all seven sachets per week for all 24 weeks. The most common reason for not taking powder in the DS group was illness, such as diarrhoea (n = 20), cough (n = 10) and forgetting to take supplements (n = 32).

The monitoring reports showed that mothers of the children in both the intervention groups reported that their children had constipation or dark stool. There was no significant difference in reports of illness (diarrhoea or cough) between the control, DS and TWS groups (32.7%, 39.1%, and 34.2%, respectively; *p *= 0.587), 42.1% (93/221) of mothers reported that sprinkles changed the colour of their children's food and 43.9% (97/221) reported that sprinkles had an unpleasant smell or taste. Some mothers mixed the sprinkles in liquids such as juice or milk. Many of the mothers felt the MMP had increased their child's appetite (31.7%) and playfulness (48.4%).

## Discussion

This randomized controlled trial was used to compare the effect of taking two sachets of MMP supplementation per week with taking a daily sachet in reducing anaemia prevalence and in improving haemoglobin concentration and growth among 331 infants and young children in rural communities of Lao PDR. The study showed that MMP supplementation was effective in reducing anaemia prevalence and in improving haemoglobin concentration in both treatment groups. This result agrees with the results of previous studies in several developing countries [17-25].

Overall, this study showed that the use of either two sachets per week or a daily sachet of MMP supplements resulted in similar increases in haemoglobin concentration and similar reductions in anaemia prevalence. This result supports similar studies in Vietnam [28], Indonesia [34] and Bangladesh[35]. We found that for severely to moderately anaemic children, daily supplementation of MMP was more effective in reducing anaemia prevalence and in improving haemoglobin concentration than TWS. This supports the results of many other micronutrients supplementation trials that suggest that daily micronutrient supplement was the best treatment for improving both anaemia and haemoglobin concentration [36-38]. The reduction of anaemia was also observed in the control group, but the reduction was relatively smaller than in the DS and TWS groups. The average increase in haemoglobin concentration in the DS and TWS groups was 18.6 and 15.6 g/L, respectively, higher than in the control group, for which the average increase was 9.3 g/L (*p *<0.001).

Several studies have shown that weekly iron supplementation, either alone or with other micronutrients, is as effective as daily iron supplementation in reducing anaemia in children [28,34]. Other studies have indicated that daily iron supplementation is more efficient [39-41]. However, daily multiple micronutrients supplementation should be better than daily iron alone for controlling anaemia, because anaemia is not caused by iron deficiency alone but by the lack of other micronutrients, such as riboflavin, folic acid, vitamin C, and vitamin A, that are known to favour iron absorption and/or haematopoiesis [42,43].

The DS group received 70 mg of iron every week, whereas the TWS group received 20 mg of iron in a week. Over the 24 weeks of the trial, 43.6% of the DS group received 1,680 mg of iron (168 sachets × 10 mg), and 27.3% of the DS group received 960 mg of iron (96 sachets × 10 mg). This amount should be sufficient to produce a haemoglobin response. The amount of iron recommended by the Sprinkles Global Health Initiative for preventing anaemia among children under five years of age is 600 mg. The DS and TWS groups both showed a significant dose-response. If a daily dose of MMP was used with full compliance, it should have been possible to eliminate anaemia in the study subjects during the 6 months of trial.

The positive effects of iron [44,45], vitamin A[46], and zinc [47] on child growth have been reported. Some studies have found that supplementation with multiple micronutrients had a positive effect on length gain, whereas in others, either weight gain improved or there was no effect[48]. We observed that inadequate breastfeeding and weaning practices might contribute to high rates of malnutrition among study subjects as for general population in these age groups. Although the young children (age less than 24 months), 39.1% were already stunted. This high rate of stunting was similar to the prevalence found among children from the same age group in a 2006 national nutrition survey [13]. Overall, during the supplementation period an improvement was observed in height-for-age Z scores among three groups, but the effect of MMP was unclear. This may not be related with MMP supplementation because the change in height-for-age was smaller in TWS group, but children in the daily group were similar to the control group. The decrease in weight for height Z scores might be because of the episodes of morbidity among study subjects during the study period. Acute episodes of morbidity might have direct and immediate impact on weight than on height. Secondly, the lack of effect of MMP on weight may have been because the complementary foods consumed by the children were inadequate in energy; no additional complementary foods were provided to the families. Thirdly, it might be related to timing. If the trial period coincides with the period of dietary transition when the diet becomes more qualitatively deficient than quantitatively deficient, the results may be compromised [49].

This study design had some limitations: (i) the anaemic children were not equally distributed among the groups; (ii) the different number of home visits for the two intervention groups may have influenced the amount of attention given to feeding practices and compliance; (iii) because of financial and technical constraints in the Lao context, it was not possible to provide a mask to the mothers or to the field workers who delivered the MMP sprinkles. However, the strength of the study is in its low dropout rate (1.5%).

The results of this trial are of relevance beyond the immediate study zone, because the nutritional status of the children in the study is similar to that of most of the children in Lao PDR. The proportion of children in the study who had stunted growth was 40.2%, and this compares well with the proportion of preschool children (40.4%) reported to have stunted growth for Lao PDR overall. Anaemia was more common in the study subjects (48.9%) compared with the national estimated anaemia prevalence of 41% in pre-school children [13].

This study indicates that the home fortification of complementary foods with multiple micronutrient supplements is an effective choice for reducing the prevalence of anaemia in children living in a setting where locally available iron-rich foods may not be affordable or accessible. The children in remote areas of Lao PDR would potentially benefit if sprinkles were incorporated into the current outreach program of the primary health care services, especially if implemented in combination with a social advocacy strategy to encourage their use to prevent anaemia. Over a longer term, health education that aims to modify food habits would be necessary to improve child growth rates.

## Conclusions

The results of this trial suggest that daily MMP supplementation produced the best result in terms of anaemia reduction. However, the results are insufficient to make recommendations to the Ministry of Health authorities about which supplementation scheme will be most appropriate to treat anaemia and multiple micronutrient deficiencies during infancy. The results are inconclusive because even in the DS group, the group for which the intervention was most effective, 32.2% of the subjects remained anaemic at the end of the trial. This study indicates that, in addition to supplementation with MMP sprinkles, long-term educational intervention to promote healthy weaning practices and the consumption of nutritionally complementary foods is necessary.

### Ethical approval

Ethical approval for this study was obtained from the Nagasaki University Ethical Review Board (Japan) and the National Ethic Committee for Health Research of the Ministry of Health (Lao PDR). A parent or guardian for all children participating in the study provided written informed consent.

## Competing interests

The authors declare that they have no competing interests.

## Authors' contributions

SK conceived of the study; NM-T, SK, TS, MH, TY, KM, and BB designed the study; SK carried out the field work; SK, TS, MH and NM-T analysed the data; NM-T, SK, TS, MH, TY, KM, and BB interpreted the data; SK and TS drafted the manuscript; MH, JO, TY, KM, NM-T, and BB revised the manuscript. All authors read and approved the final manuscript.

## References

[B1] WHO/WFP/UNICEF Preventing and Controlling Micronutrient Deficiencies in Population Affected by an Emergency: Multiple vitamins and minerals supplements for pregnant and lactating women, and for children aged 6-59 mo Joint statement by the WHO/WFP/UNICEF WHO Geneva 2007

[B2] Kikafunda JK Walker AF Collett D Tumwine JK Risk factors for early childhood malnutrition in Uganda Pediatrics 1998 102 4 E45. 9755282 10.1542/peds.102.4.e45

[B3] Vazir S Naidu AN Vidyasagar P Nutritional status, psychosocial development and the home environment of Indian rural children Indian Pediatr 1998 35 10 959 966 10216718

[B4] Idjradinata P Pollitt E Reversal of developmental delays in iron-deficient anaemic infants treated with iron Lancet 1993 341 8836 1 4 10.1016/0140-6736(93)92477-B 7678046

[B5] Rahmathullah L Underwood BA Thulasiraj RD Milton RC Ramaswamy K Rahmathullah R Babu G Reduced mortality among children in southern India receiving a small weekly dose of vitamin A N Engl J Med 1990 323 14 929 935 10.1056/NEJM199010043231401 2205798

[B6] Brown KH Peerson JM Allen LH Effect of zinc supplementation on children's growth: a meta-analysis of intervention trials Bibl Nutr Dieta 1998 54 76 83 9597173 10.1159/000059448

[B7] Castillo-Duran C Rodriguez A Venegas G Alvarez P Icaza G Zinc supplementation and growth of infants born small for gestational age J Pediatr 1995 127 2 206 211 10.1016/S0022-3476(95)70296-2 7636643

[B8] Umeta M West CE Haidar J Deurenberg P Hautvast JG Zinc supplementation and stunted infants in Ethiopia: a randomised controlled trial Lancet 2000 355 9220 2021 2026 10.1016/S0140-6736(00)02348-5 10885352

[B9] de Pee S Richard D Semba, Martin W Bloem Need, Efficacy, and Effectiveness of Multiple Vitamin/Mineral Supplements for Young Children and Considerations for Programs Nutrition and Health in Developing Countries 2008 second Humana Press. Totowa, NJ

[B10] Gibson RS Ferguson EL Lehrfeld J Complementary foods for infants feeding in developing countries: Their nutrient adequacy and improvement Eur J Clin Nutr 1998 52 764 770 10.1038/sj.ejcn.1600645 9805226

[B11] Allen LH Causes of nutrition-related public health problems of preschool children: available diet J Pediatr Gastroenterol Nutr 2006 43 Suppl 3 S8 12 10.1097/01.mpg.0000255845.99905.20 17204983

[B12] Miyoshi M Phommasack B Nakamura S Kuroiwa C Nutritional status of children in rural Lao PDR: who are the most vulnerable? Eur J Clin Nutr 2005 59 7 887 890 10.1038/sj.ejcn.1602160 15915154

[B13] NSC Monitoring the situation of children and women: Multiple Indicators Cluster Survey and National Nutrition Survey. UNICEF/MOH/MPI Lao PDR. 2006 Final Report 2006

[B14] UNICEF/UNU/WHO Iron deficiency anemia: assessment, prevention, and control. A guide for programme managers WHO/NIID [Report no013] 2001

[B15] Barennes H Simmala C Odermatt P Thaybouavone T Vallee J Martinez-Aussel B Newton PN Strobel M Postpartum traditions and nutrition practices among urban Lao women and their infants in Vientiane, Lao PDR Eur J Clin Nutr 2009 63 3 323 331 10.1038/sj.ejcn.1602928 18000519 PMC3435433

[B16] Zlotkin S Antwi KY Schauer C Yeung G Use of microencapsulated iron(II) fumarate sprinkles to prevent recurrence of anaemia in infants and young children at high risk Bull World Health Organ 2003 81 2 108 115 12756979 PMC2572396

[B17] Hirve S Bhave S Bavdekar A Naik S Pandit A Schauer C Christofides A Hyder Z Zlotkin S Low dose 'Sprinkles'-- an innovative approach to treat iron deficiency anemia in infants and young children Indian Pediatr 2007 44 2 91 100 17351300

[B18] Adu-Afarwuah S Lartey A Brown KH Zlotkin S Briend A Dewey KG Home fortification of complementary foods with micronutrient supplements is well accepted and has positive effects on infant iron status in Ghana Am J Clin Nutr 2008 87 4 929 938 18400716 10.1093/ajcn/87.4.929

[B19] Menon P Ruel MT Loechl CU Arimond M Habicht JP Pelto G Michaud L Micronutrient Sprinkles reduce anemia among 9- to 24-mo-old children when delivered through an integrated health and nutrition program in rural Haiti J Nutr 2007 137 4 1023 1030 17374671 10.1093/jn/137.4.1023

[B20] Zlotkin S Arthur P Antwi KY Yeung G Treatment of anemia with microencapsulated ferrous fumarate plus ascorbic acid supplied as sprinkles to complementary (weaning) foods Am J Clin Nutr 2001 74 6 791 795 11722961 10.1093/ajcn/74.6.791

[B21] Zlotkin S Arthur P Schauer C Antwi KY Yeung G Piekarz A Home-fortification with iron and zinc sprinkles or iron sprinkles alone successfully treats anemia in infants and young children J Nutr 2003 133 4 1075 1080 12672922 10.1093/jn/133.4.1075

[B22] Christofides A Asante KP Schauer C Sharieff W Owusu-Agyei S Zlotkin S Multi-micronutrient Sprinkles including a low dose of iron provided as microencapsulated ferrous fumarate improves haematologic indices in anaemic children: a randomized clinical trial Matern Child Nutr 2006 2 3 169 180 10.1111/j.1740-8709.2006.00060.x 16881929 PMC6860742

[B23] Giovannini M Sala D Usuelli M Livio L Francescato G Braga M Radaelli G Riva E Double-blind, placebo-controlled trial comparing effects of supplementation with two different combinations of micronutrients delivered as sprinkles on growth, anemia, and iron deficiency in cambodian infants J Pediatr Gastroenterol Nutr 2006 42 3 306 312 10.1097/01.mpg.0000189363.07040.4b 16540800

[B24] Zlotkin SH Schauer C Christofides A Sharieff W Tondeur MC Hyder SM Micronutrient sprinkles to control childhood anaemia PLoS Med 2005 2 1 e1 10.1371/journal.pmed.0020001 15696200 PMC545194

[B25] Zlotkin SH Christofides AL Hyder SM Schauer CS Tondeur MC Sharieff W Controlling iron deficiency anemia through the use of home-fortified complementary foods Indian J Pediatr 2004 71 11 1015 1019 10.1007/BF02828118 15572823

[B26] Zlotkin S Micronutrient sprinkles for use in infants and young children: guidelines on recommendations for use and program monitoring and evaluation Sprinkles Global Health Initiative 2008

[B27] Mora JO Iron supplementation: overcoming technical and practical barriers J Nutr 2002 132 4 Suppl 853S 855S 11925496 10.1093/jn/132.4.853S

[B28] Thu BD Schultink W Dillon D Gross R Leswara ND Khoi HH Effect of daily and weekly micronutrient supplementation on micronutrient deficiencies and growth in young Vietnamese children Am J Clin Nutr 1999 69 1 80 86 9925127 10.1093/ajcn/69.1.80

[B29] Murayama N Natsuhara K Sasaki S Kounnavong S Phonglusa K Sithideth D Dietary intake and indicators of dietary change in Lahanam, Savannakhet, Lao People's Democratic Republic in Health Development Study in Lahanam, Savannakhet, Lao P.D.R April 2004 to March 2006. Project Report 2006

[B30] Drewett R Amatayakul K Wongsawasdii L Mangklabruks A Ruckpaopunt S Ruangyuttikarn C Baum D Imong S Jackson D Woolridge M Nursing frequency and the energy intake from breast milk and supplementary food in a rural Thai population: a longitudinal study Eur J Clin Nutr 1993 47 12 880 891 8156985

[B31] Cohen ARS-FJ HemoCue system for haemoglobin measurement. Evaluation in anaemic and non-anaemic children Am J Clin Pathol 1988 90 302 305 3414603 10.1093/ajcp/90.3.302

[B32] de Onis M Onyango AW Van den Broeck J Chumlea WC R M The WHO Multicentre Growth Reference Study Group. Measurement and standardization protocols for anthropometry used in the construction of a new international growth reference Food and Nutrition Bulletin 2004 25 Supplement1 S27 36 15069917 10.1177/15648265040251S104

[B33] WHO WHO Anthro for personal computers, version 3.01: Software for assessing growth and development of the world's children 2009

[B34] Schultink W Gross R Gliwitzki M Karyadi D Matulessi P Effect of daily vs twice weekly iron supplementation in Indonesian preschool children with low iron status Am J Clin Nutr 1995 61 1 111 115 7825521 10.1093/ajcn/61.1.111

[B35] Hyder SMZ Haseen F Rahman M Tondeur MC aZS H Effect of daily versus once-weekly home fortification with micronutrient Sprinkles on hemoglobin and iron status among young children in rural Bangladesh Food and Nutrition Bulletin 2007 28 2 156 164 10.1177/156482650702800204 24683674

[B36] Smuts CM Dhansay MA Faber M van Stuijvenberg ME Swanevelder S Gross R Benade AJ Efficacy of multiple micronutrient supplementation for improving anemia, micronutrient status, and growth in South African infants J Nutr 2005 135 3 653S 659S 15735110 10.1093/jn/135.3.653S

[B37] Untoro J Karyadi E Wibowo L Erhardt MW Gross R Multiple micronutrient supplements improve micronutrient status and anemia but not growth and morbidity of Indonesian infants: a randomized, double-blind, placebo-controlled trial J Nutr 2005 135 3 639S 645S 15735108 10.1093/jn/135.3.639S

[B38] Wijaya-Erhardt M Erhardt JG Untoro J Karyadi E Wibowo L Gross R Effect of daily or weekly multiple-micronutrient and iron foodlike tablets on body iron stores of Indonesian infants aged 6-12 mo: a double-blind, randomized, placebo-controlled trial Am J Clin Nutr 2007 86 6 1680 1686 18065586 10.1093/ajcn/86.5.1680

[B39] Zavaleta N Respicio G Garcia T Efficacy and acceptability of two iron supplementation schedules in adolescent school girls in Lima, Peru J Nutr 2000 130 2S Suppl 462S 464S 10721929 10.1093/jn/130.2.462S

[B40] Sungthong R Mo-Suwan L Chongsuvivatwong V Geater AF Once weekly is superior to daily iron supplementation on height gain but not on hematological improvement among schoolchildren in Thailand J Nutr 2002 132 3 418 422 11880565 10.1093/jn/132.3.418

[B41] Hop le T Berger J Multiple micronutrient supplementation improves anemia, micronutrient nutrient status, and growth of Vietnamese infants: double-blind, randomized, placebo-controlled trial J Nutr 2005 135 3 660S 665S 15735111 10.1093/jn/135.3.660S

[B42] Mejia LA Chew F Hematological effect of supplementing anemic children with vitamin A alone and in combination with iron Am J Clin Nutr 1988 48 3 595 600 3414574 10.1093/ajcn/48.3.595

[B43] Fishman SM Christian P West KP The role of vitamins in the prevention and control of anaemia Public Health Nutr 2000 3 2 125 150 10948381 10.1017/s1368980000000173

[B44] Angeles IT Schultink WJ Matulessi P Gross R Sastroamidjojo S Decreased rate of stunting among anemic Indonesian preschool children through iron supplementation Am J Clin Nutr 1993 58 3 339 342 8237843 10.1093/ajcn/58.3.339

[B45] Lawless JW Latham MC Stephenson LS Kinoti SN Pertet AM Iron supplementation improves appetite and growth in anemic Kenyan primary school children J Nutr 1994 124 5 645 654 8169656 10.1093/jn/124.5.645

[B46] Murdiana A Azis I Saidin S Jahari AB Karyadi D Vitamin A-fortified monosodium glutamate and vitamin A status: a controlled field trial Am J Clin Nutr 1988 48 5 1265 1270 3189215 10.1093/ajcn/48.5.1265

[B47] Hambidge KM Zinc deficiency in young children Am J Clin Nutr 1997 65 1 160 161 8988929 10.1093/ajcn/65.1.160

[B48] Chhagan MK Van den Broeck J Luabeya KK Mpontshane N Tomkins A Bennish ML Effect on longitudinal growth and anemia of zinc or multiple micronutrients added to vitamin A: a randomized controlled trial in children aged 6-24 months BMC Public Health 2010 10 145 10.1186/1471-2458-10-145 20298571 PMC2847544

[B49] Allen LH Interventions for micronutrient deficiency control in developing countries: past, present and future J Nutr 2003 133 11 Suppl 2 3875S 3878S 14672284 10.1093/jn/133.11.3875S

